# A Robust Head Tracking System Based on Monocular Vision and Planar Templates

**DOI:** 10.3390/s91108924

**Published:** 2009-11-11

**Authors:** Fernando Caballero, Iván Maza, Roberto Molina, David Esteban, Aníbal Ollero

**Affiliations:** 1 Robotics, Vision and Control Group, University of Seville, Avd. de los Descubrimientos s/n, 41092, Sevilla, Spain; E-Mail: imaza@cartuja.us.es; 2 Boeing Research ' Technology Europe, Canada Real de las Merinas, 1-3, Bldg 4, 28042 Madrid, Spain; E-Mails: roberto.molina@boeing.com (R.M.); david.esteban-campillo@boeing.com (D.E.); 3 Center for Advanced Aerospace Technology (CATEC), Parque Tecnológico y Aeronáutico de Andalucía, C. Wilbur y Orville Wright 17-19-21, 41309, La Rinconada, Spain; E-Mail: aollero@cartuja.us.es

**Keywords:** head tracking, computer vision, homography

## Abstract

This paper details the implementation of a head tracking system suitable for its use in teleoperation stations or control centers, taking into account the limitations and constraints usually associated to those environments. The paper discusses and justifies the selection of the different methods and sensors to build the head tracking system, detailing also the processing steps of the system in operation. A prototype to validate the proposed approach is also presented along with several tests in a real environment with promising results.

## 1. Introduction

Latest advances in technology and the growing computational capabilities of desktop computers have allowed the introduction of new devices for human-machine interaction. These devices usually provide functionalities to substitute the computer mouse. In the last years, new devices to estimate the real position of the user's head in real time under certain constraints have been developed.

Most of these devices rely on cameras and image processing algorithms. In general, they can be divided into two main application areas: 2DoF (Degrees of Freedom) and 6DoF head tracking. The 2DoF tracking products are focused on mouse emulation. They replace the standard computer mouse for people who cannot use their hands when controlling a computer or an augmentative communication device. On the other hand, 6DoF tracking is mainly oriented to gaming, allowing complete user immersion into different computer games.

**Figure 1.**
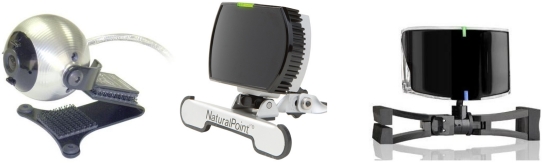
*Left*: Tracker Pro (Madentec). *Center*: SmartNav 4 AT (NaturalPoint). *Right*: TrackIR 4 (NaturalPoint).

2DoF head tracking applications and products are easy to find. Most of these products are based on image processing and marks/spots placed in the users' head (on a hat for instance). They also provide the two angles information used to move the mouse left/right and up/down. *Tracker Pro* [1] (see Figure 1) is a good example. This product is based on an USB camera and a software package. It is very reliable and has a wide field of view (about 45 degrees) and supports sunlight compatibility. Other examples are the *Headmouse Extreme* [2] or the *SmartNav 4 AT* [3] (see Figure 1).

6DoF head tracking moves one step forward and allows estimating the complete position and orientation of the user's head in real time. Most of the approaches can be divided into two groups: based on human face detection and based on visual pattern detection, both using image processing. In the first group, several research works [4–6] have shown robust estimation processing stages of the position and orientation of the user's head. In those cases, model-based head detection is used to initialize the tracker and also to estimate the 6DoF localization of the head. The main concern is usually related to the reliability of the face detection stage. Actually, many recent works have been devoted to increase the robustness of this kind of approaches. For instance, in [7] particle filtering and complex tracking policies are used to implement a robust system.

The second group of 6DoF head tracking approaches makes use of some patterns/marks that allow simplifying the head detection process in the sequence of images. Thus, [8] uses infrared LEDs mounted on the user's head to localize it. A different approach is applied in [9], where the camera is mounted on the user's head and some landmarks are detected and used to localize the head. Nowadays, the commercial products are mainly focused in this kind of approaches and normally make use of camera and visual/IR patterns mounted on the head. *TrackIr* [10] is a good example of these systems (see Figure 1). It uses a 3D pattern visible in the infrared band to estimate the position and orientation of the user.

**Figure 2.**
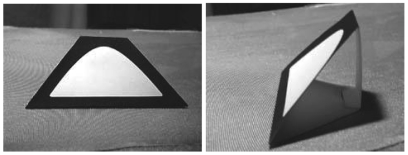
Visual pattern used in the Cachya head tracking system.

In this paper, a new 6DoF head tracking system able to provide real time position and orientation of the head, minimizing the interferences with the user operations, is proposed. This is an aspect that differentiates our work from the above presented approaches. Thus, the previously introduced 6DoF products require a 3D pattern mounted on the user's head, as shown in Figure 2. They are normally attached to a hat that the user wears. Although it is common for gamers, operators are subject to hard constraints in terms of additional devices, i.e., they must be compatible with current systems like headphones, haptic systems, etc.

The design guidelines were focused on integration and robustness. To fulfill such constraints, the following system was proposed:

- A head tracking system based on the localization of an infrared pattern that the operator carries on the head. The reason to use infrared emission is that it is out of the visible band, so it is not perceived by the operator. Moreover, it is possible to use infrared filters to remove visual information, remaining only the infrared information and simplifying the pattern detection algorithms.
- The infrared pattern is integrated into the headphones used by the operator in order to avoid disturbing his working environment. Thus, the pattern is 2D and not 3D as usual in the previously described products and approaches.

To the best of our knowledge, this is one of the first implementations of a 6DoF head-tracking system based on planar templates. The research is focused on a particular problem: the head-tracking in teleoperation stations. This environment poses very hard constraints in terms of robustness, usability and compatibility with already existing devices:

- Usability and compatibility are addressed by means of the proposed prototype based on an infrared planar template integrated into the user's headphones.
- Robustness is explicitly addressed in the approach by including marker tracking in the image space. This feature makes a difference with respect to the commercial devices in which environment disturbances such as sun light, reflections, halogen lamps or IR remotes have a direct impact in the head-tracking estimation. The proposed tracking method allows rejecting such disturbances once the pattern has been detected. In addition, it allows decreasing the computational requirements for image processing because the filter prediction bounds the area in which the markers should be projected and hence, the processing can be applied only locally.

The paper is structured as follows: Section 2. details the design of the head-tracking system and the image processing algorithms used to compute the user's head position and orientation. Later, Section 3. describes the prototype that has been implemented to test the proposed approach. This prototype is used in Section 4. in two different experiments to validate the system. Finally, Section 5. presents the conclusions and future developments.

## 2. Head Tracking System

**Figure 3.**
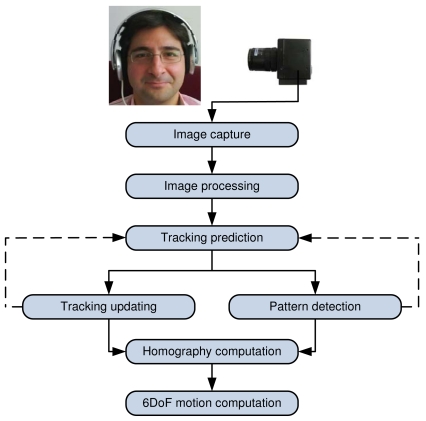
Different stages involved in the proposed head tracking system.

This section details the design, taking into account several practical issues. The proposed head tracking process can be decomposed into the steps shown in Figure 3. First, an image of the environment is captured and processed by the system to prepare the detection of the infrared pattern. Then, the pattern is searched using two possible approaches: pattern detection or tracking considering previous information. The normal operation of the system will be to track the position of the pattern. If the tracking fails or there is not enough information to compute the tracking, then the system will try to detect it again.

Once the infrared pattern is detected in the image, the system will compute the homography matrix that relates the pattern and its projection, and this homography will be decomposed into the real position and orientation of the user's head.

The processing carried out in each step is further detailed in the next sections.

### 2.1. Image Capture

All the head tracking software developed is operating system independent up to the image capture level. For this purpose the libdc1394 library for Linux has been used. This library provides a complete set of functions to manage any firewire camera that implements the DCAM protocol for machine vision, from simple image capture to camera parametrization (shutter, exposure, gain, etc.).

Thus, the firewire cameras' ability in setting up image capture parameters such as gain, shutter or iris allows implementing methods for camera self-configuration, making the system much more robust to changes in the lighting conditions.

The firewire image capture library is used in the software to capture images and to setup the following parameters: brightness, exposure, gamma, shutter and gain. All of them are set to zero in order to manually manage any image processing, so that our software can take the control of the entire image domain.

Regarding camera synchronization, an external digital signal can be used as the trigger to ensure the image timing. However, given the low latency of the firewire bus triggering (less than a microsecond) and assuming a static camera (which is the case), the firewire internal triggering is used in the implementation in order to simplify the camera setup. The camera is configured to capture images at 30 Hz, so the system will provide head tracking information every 33 ms.

### 2.2. Image Processing

The image processing stage is probably the most sensitive, but simplest stage, in the head tracking system. The goal of this stage is to process the image in order to detect the set of bright LEDs. This problem is particularly complex in the sense that depends on the camera environment.

As it was mentioned above, the camera integrates an infrared filter to suppress visible information from the image and pass the infrared pattern. However, infrared is present in many environments: daylight, lamps or infrared communication. The system is designed to be able to eliminate part of the disturbances induced by the environment.

**Figure 4.**
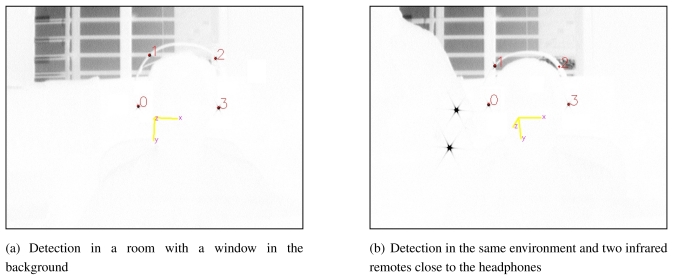
Images showing the detection process during the operation of the system. The four infrared LEDs detected are marked by red dots over the raw images captured by the camera (notice that the colors are inverted). The computed coordinate frame is also shown as an overlay on the image captured. (a) Detection in a room with a window in the background (b) Detection in the same environment and two infrared remotes close to the headphones

Then, the camera, the filter and the infrared LEDs have to be carefully selected to obtain maximum gain into the bandwidth of interest (this selection will be discussed in Section 3.). In these conditions, the images captured by the camera are similar to the those presented in Figure 4. Four black spots, that represent the four emitting LEDs of the pattern, can be found (notice that the colors are inverted). Additional infrared information is also present in the images: sunlight from a window in the background and two ir-Ir remotes.

The infrared detection is based on finding four maximums into the image (each maximum corresponding to an infrared LED). Additionally, these maximums will be subject to the following constraints:

- The grayscale of each maximum must be greater than a given threshold. Assuming that the infrared information provided by the LEDs is always greater than the infrared present in the environment, this threshold helps to separate between LED information and noise from the environment. In the current implementation, this threshold is set to 40 (15% of the maximum value that can be perceived by the camera). If there are no enough maximums greater than this threshold, the system drop off the image and cancel the head tracking estimation with that image.
- In a general case, the LED will be projected in the image as an ellipse, but not as a single pixel. The ellipse of each detected peak must have a minimal and maximal area. This information can be used to detect and eliminate potential outliers, allowing to reject mismatches produced by reflections or noise. However, given the elliptical nature of the detected peaks, the position of the LED must be computed as the centroid of such ellipse. Then, the position [*C_u_, C_v_*]*^t^* of the LED is finally given by
  (1) $$C_{u}=1/N\sum_{k=0}^{N}p_{u},C_{v}=1/N\sum_{k=0}^{N}p_{v},$$
  where *N* is the number of pixels that compose the ellipse, and *p_u_* and *p_v_* stand for the pixel column and row respectively.
- The distance among maximums must be greater than a given threshold. The idea behind this constraint is to avoid the selection of maximums too close to each other. This is very usual when the infrared emission is split due to the presence of objects such as hair, glasses, etc. This threshold has been set to 60 pixels. Notice that this threshold limits the distance at which the user can be located with respect to the camera. The current value allows standing at more than two meters from the camera.

These constraints are applied sequentially in the above order. Thus, a group of potential maximums from the first step will be obtained; they will be cut off depending on the size of the projected ellipse in the image and, finally, this sub-group of maximums is reduced to four taking into account the minimal distance constraint.

Figure 4 shows the result of applying this algorithm to different images. The four LEDs of the pattern (red spots) are detected and many outliers are rejected by the algorithm.

### 2.3. Infrared Pattern Matching

The previous step provides a set of points in the image that may correspond with the LEDs of the infrared pattern. This set of matches was filtered according to geometrical and gray scale constraints. However, the match between each LED and its projection in the image is still unknown. This section details how to match the infrared pattern with its projection in the image.

**Figure 5.**
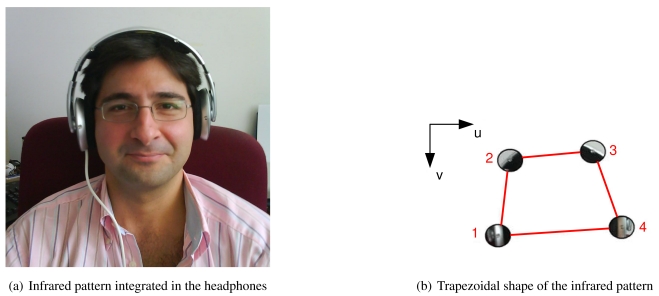
Shape of the infrared pattern integrated in the headphones. (a) Infrared pattern integrated in the headphones (b) Trapezoidal shape of the infrared pattern

The matching between LEDs and projections is based on the geometrical constraints imposed by the pattern. Our pattern is composed of four LEDs mounted into the headphones of the operator, and has a trapezoidal shape (see Figure 5), i.e., the bottom LEDs (labelled as 1 and 4) are more separated than the two LEDs in the top (labelled as 2 and 3). This characteristic allows to easily sort out between bottom and top LEDs based on the relative distances.

Then, given the set of projections p*_i_* = [*u_i_, v_i_*]*^t^, i* = 1, …, 4, where *u_i_* holds for the rows and *v_i_* for the columns with the zero in the upper left corner of the image (see Figure 5), the first step consists on sorting the projections depending on the value of *v_i_* from maximal to minimal values. Then, assuming that the pattern is rotated less than forty five degrees, the two first projections correspond to the bottom LEDs (labelled as 1 and 4 in Figure 5) and the other two to the upper projections (labelled as 2 and 3). Finally, it is possible to distinguish between the left and right projections depending on the value of *u_i_*.

Later, constraints in *u_i_* are used to verify the detection. Thus, given the matching between projections and LEDs in the pattern, and assuming a rotation less than forty five degrees, the projections belonging to LEDs 1 and 2 must have an *u_i_* coordinate shorter than projections 3 and 4.

Then, this method is valid only when the pattern is rotated less than fortyfive degrees and tilted less than ninety degrees. It is easy to see that the LED/projection association is undetermined out of these limits. Nevertheless, this is not a hard constraint in the system because the operators's head will be normally within such limits. In addition, this limitation holds for the detection stage, but not for the tracking process detailed in next section.

### 2.4. Pattern Tracking in the Image Plane

Now, there is an initial estimation about the projection of the pattern into the camera and the next step is to track the position of these projections during the images sequence. The tracking of the projections allows to improve the image processing and pattern detection thanks to the prediction phase. Thus, the predicted position of the projections will be used by the whole head tracking system to reject outliers and decrease the searching area in the image processing stage.

Then, the tracking process is divided into two basic parts: prediction and updating. The first step consists on predicting the position of the projections in the image and the second uses the current position of the projections (obtained by means of all the previous algorithms) to update the predictions.

It is proposed a Kalman Filter for each projection tracking. The filter will estimate the position and velocity of each projection in the image, whereas the covariance matrix associated to the projections will determine the searching areas and the candidates that can be used as projections. An independent Kalman Filter will be launched for each projection, being the state vector for projection *i* the following:

(2) $${\text{x}}_{i}={\left[{\text{p}}_{i},{\text{v}}_{i}\right]}^{t}$$

where p*_i_* and v*_i_* are the position and velocity of the projection *i* in the current image expressed in pixels and pixels/s respectively.

During the prediction stage, it will be assumed that each Δ*t* seconds, an instant perturbation in the velocity of the pixels will be produced (Δv holds for this perturbation). It will be assumed that the components of this velocity are independent Gaussians with zero mean and known standard deviation, so:

(3) $$\Delta \text{v}=\left[\begin{array}{c} \Delta v_{u}\\ \Delta v_{v} \end{array}\right]=\left[\begin{array}{c} \mathbb{N}(0,\sigma_{u}^{2})\\ \mathbb{N}(0,\sigma_{v}^{2}) \end{array}\right].$$

Then, the prediction model for the projection *i*, from time instant *k* − 1 to *k* is given by

(4) $$\left[\begin{array}{c} {\text{p}}_{i}(k)\\ {\text{v}}_{i}(k) \end{array}\right]=\left[\begin{array}{c} {\text{p}}_{i}(k-1)+({\text{v}}_{i}(k-1)+\Delta \text{v})\Delta t\\ {\text{v}}_{i}(k-1)+\Delta \text{v} \end{array}\right].$$

The Kalman filter proposes the following equations to predict the new state and its covariance matrix:

(5) $${\text{x}}_{i}^{-}(k)={\text{A}}_{i}{\text{x}}_{i}(k-1)+{\text{B}}_{i}{\text{u}}_{i}(k-1)$$

(6) $${\text{P}}_{i}^{-}(k)={\text{A}}_{i}{\text{P}}_{i}(k-1){\text{A}}_{i}^{T}+{\text{Q}}_{i}$$

By comparing with (4), it is easy to identify the matrices **A***_i_*, **B***_i_* and **Q***_i_* as

(7) $${\text{A}}_{i}=\left[\begin{array}{cccc} 1 & 0 & \Delta t & 0\\ 0 & 1 & 0 & \Delta t\\ 0 & 0 & 1 & 0\\ 0 & 0 & 0 & 1 \end{array}\right],{\text{B}}_{i}=0,{\text{Q}}_{i}=\left[\begin{array}{cccc} {\left(\Delta t\sigma_{u}\right)}^{2} & 0 & \Delta t\sigma_{u}^{2} & 0\\ 0 & {\left(\Delta t\sigma_{v}\right)}^{2} & 0 & \Delta t\sigma_{v}^{2}\\ \Delta t\sigma_{u}^{2} & 0 & \sigma_{u}^{2} & 0\\ 0 & \Delta t\sigma_{v}^{2} & 0 & \sigma_{u}^{2} \end{array}\right].$$

In the system implementation, the value of Δ*t* is dynamically computed during the program execution. A timer is launched when the filter updating is done and stopped when the filter prediction is computed, being Δ*t* the elapsed time. This value is usually in the order of 0.33 ms.

Another important part in the prediction is the value estimation of *σ_u_* and *σ_v_*. In order to have a balance between conservativeness and efficiency in the prediction, this value is composed of two terms: a constant and a term that depends on the current filter state. Thus, such values are computed as follows:

(8) $$\sigma_{u}=1+0.2{\hat{v}}_{u}(k-1)$$

(9) $$\sigma_{v}=1+0.2{\hat{v}}_{v}(k-1)$$

On the other hand, the updating model considers the measurement provided by the detection algorithms. Then, the estimated position of the projection *i* will be directly mapped into the state vector x*_i_*. If *z_i_(k)* is the measurement corresponding to the *i*-th projection, the updating equation can be expressed as

(10) $${\text{z}}_{i}(k)={\text{p}}_{i}(k).$$

The new measurement *z_i_(k)* is considered in the Kalman Filter by means of the following equations:

(11) $${\text{K}}_{i}(k)={\text{P}}_{i}^{-}(k){\text{H}}_{i}^{T}{\left({\text{H}}_{i}{\text{P}}_{i}^{-}(k){\text{H}}_{i}^{T}+{\text{R}}_{i}\right)}^{-1}$$

(12) $${\text{x}}_{i}(k)={\text{x}}_{i}^{-}(k)+{\text{K}}_{i}(k)({\text{z}}_{i}(k)-{\text{H}}_{i}{\text{x}}_{i}^{-}(k))$$

(13) $${\text{P}}_{i}(k)=(\text{I}-{\text{K}}_{i}(k){\text{H}}_{i}){\text{P}}_{i}^{-}(k)$$

Considering (10), the following updating matrices can be derived:

(14) $${\text{H}}_{i}=\left[\begin{array}{cccc} 1 & 0 & 0 & 0\\ 0 & 1 & 0 & 0 \end{array}\right],{\text{R}}_{i}=\left[\begin{array}{cc} \sigma_{iu}^{2} & 0\\ 0 & \sigma_{iv}^{2} \end{array}\right]$$

where $\sigma_{iu}^{2}$ and $\sigma_{iv}^{2}$ stand for the variance associated to the detected projection *i*. Assuming that every projection includes a standard deviation of 2 pixels in the detection due to image saturation and binarization, this leads to $\sigma_{iu}^{2}=4$ and $\sigma_{iv}^{2}=4$.

### 2.5. 6DoF Pose Estimation

This section details the computation of the user's head position and orientation by using the projection provided by the tracking system. Two basic steps are carried out: first, the mathematical model that relates the projection with the infrared pattern is computed (this model is a homography), and then, the computed homography is decomposed into rotation and translation.

The following notation will be used: A 2D point in the image plane is denoted by **m** = [*u, v*]*^t^* and a 3D point in the world system reference is denoted by **g** = [*x, y, z*]*^t^*. This system reference is the frame in which the position of the infrared pattern will be finally expressed. In the case of the application presented in the paper, this frame is attached to the main screen of the operation station. It will be used the symbol ∼ to denote the augmented homogeneous vector generated by adding 1 as the last element: **m̃**= [*u,v,1*]*^t^* and **g̃** = [*x,y,z,1*]*^t^*.

#### Homography Computation

Knowing the matching between pattern and image projections, it is necessary to fit them a motion model, minimizing the error. The model used is the homography. Thus, assuming that the pattern is planar (which is our case), the projection in the image is related with the pattern through the following expression:

(15) $$k{\tilde{\text{m}}}^{\prime }=\text{H}\tilde{\text{m}}\;\text{with}\;{\tilde{\text{m}}}^{\prime }=\left[\begin{array}{c} u^{\prime }\\ v^{\prime }\\ 1 \end{array}\right],\tilde{\text{m}}=\left[\begin{array}{c} u\\ v\\ 1 \end{array}\right]\;\text{and}\;\text{H}=\left[\begin{array}{ccc} h_{11} & h_{12} & h_{13}\\ h_{21} & h_{22} & h_{23}\\ h_{31} & h_{32} & h_{33} \end{array}\right],$$

where **H** is a 3*x*3 non-singular matrix called homography and which is defined up to a scale factor *k*. This means that the homography matrix depends on 8 parameters. Four correspondences are needed to determine one homography because each correspondence gives two equations to solve the system.

If a set of four matches between the pattern and the image are given by the image processing algorithms, the following equations can be extracted for each match:

(16) $$(h_{31}u+h_{32}v+h_{33})u^{\prime }=h_{11}u+h_{12}v+h_{13}$$

(17) $$(h_{31}u+h_{32}v+h_{33})v^{\prime }=h_{21}u+h_{22}v+h_{23}$$

and reordering the expression we have

(18) $$\left[\begin{array}{ccccccccc} u & v & 1 & 0 & 0 & 0 & -uu^{\prime } & -vu^{\prime } & -u^{\prime }\\ 0 & 0 & 0 & u & v & 1 & -uv^{\prime } & -vv^{\prime } & -v^{\prime } \end{array}\right]\;\left[\begin{array}{c} h_{11}\\ h_{12}\\ h_{13}\\ h_{21}\\ h_{22}\\ h_{23}\\ h_{31}\\ h_{32}\\ h_{33} \end{array}\right]=\left[\begin{array}{c} 0\\ 0 \end{array}\right].$$

Finally, it is possible to stack the two equations provided by each projection, building a 9 × 9 matrix **A**, and compute the parameters of the homography **H**. However, it is important to note that the corresponding system of equations is homogeneous, so it cannot be solved using the classical least squares approach. In this case, the solution to the system of equations is given by the right singular vector of **A** associated with the smallest singular value. Further details can be found in [11] and [12].

#### Motion Estimation

If the camera is modelled by the usual pinhole model, the relationship between the augmented homogeneous vectors of a 3D point **g̃** and its image projection **m̃** is given by:

(19) $$k\tilde{\text{m}}=\text{C}[\text{R}|\text{t}]\tilde{\text{g}}=\text{C}[{\text{r}}_{1},{\text{r}}_{2},{\text{r}}_{3},\text{t}]\tilde{\text{g}}$$

where *k* is an arbitrary scale factor, matrix [**R**∣**t**] (called the extrinsic parameters) contains the rotation matrix **R** = [**r**_1_,**r**_2_,**r**_3_] and the translation vector **t** which relates the world coordinate system to the camera coordinate system, and **C** is the camera calibration matrix.

Without loss of generality, it is assumed that the pattern plane is on *z* = 0 of the world coordinate system, allowing us to derive the following expression:

(20) $$k\left[\begin{array}{c} u\\ v\\ 1 \end{array}\right]=\text{C}[{\text{r}}_{1},{\text{r}}_{2},{\text{r}}_{3},\text{t}]\left[\begin{array}{c} x\\ y\\ 0\\ 1 \end{array}\right]=\text{C}[{\text{r}}_{1},{\text{r}}_{2},\text{t}]\left[\begin{array}{c} x\\ y\\ 1 \end{array}\right].$$

In turn, it can be assumed that **g̃** = [*x, y*, 1]*^t^* while **r**_3_ is computed as the cross product of the computed **r**_1_ and **r**_2_. Therefore, a pattern point **g̃** and its projection **m̃** are related by a homography **H** according to

(21) $$k\tilde{\text{m}}=\text{H}\tilde{\text{g}}\;\text{with}\;\text{H}=\text{C}[{\text{r}}_{1},{\text{r}}_{2},\text{t}].$$

Then, if the homography **H** that relates the infrared pattern and the projection into the camera is known, it is possible to recover the full rotation and translation of the pattern with respect to the camera. Thus, it can be easily computed from

(22) $$\text{H}=[{\text{h}}_{1},{\text{h}}_{2},{\text{h}}_{3}]$$

(23) $${\text{r}}_{1}=\lambda {\text{C}}^{-1}{\text{h}}_{1}$$

(24) $${\text{r}}_{2}=\lambda {\text{C}}^{-1}{\text{h}}_{2}$$

(25) $${\text{r}}_{3}={\text{r}}_{1}\times {\text{r}}_{2}$$

(26) $$\text{t}=\lambda {\text{C}}^{-1}{\text{h}}_{3}$$

with *λ* = 1/‖**C**^−1^**h**_1_‖ = 1/‖**C**^−1^**h**_2_‖. In general, due to the noise in the data, the so-computed matrix **R** = [**r**_1_, **r**_2_, **r**_3_] does not satisfy the properties of a rotation matrix. It can be demonstrated that the closest (in mean squares terms) rotation matrix to the above solution will be determined by **R** = **UV***^t^*, where **U** and **V** come from the singular value decomposition of the above estimation [**r**_1_, **r**_2_, **r**_3_] = **USV***^t^*.

Further mathematical details and demonstrations can be found in [13].

## 3. Prototype Implementation

This section details the physical implementation of the head tracking system prototype. This prototype has been used to validate the previous algorithms in real time with real data gathered in the laboratory. The prototype can be divided into four basic sub-systems:

- Infrared LEDs. A total of four LEDs will be used to compose the infrared pattern that will be later detected in the images captured by the camera.
- Headphones. The infrared LEDs are mounted on the operator's headphones.
- Visual camera. It will provide the images with the infrared pattern.
- Infrared filter. Specially designed for the infrared LEDs, it allows to filter visual information.

### 3.1. Infrared LEDs

**Figure 6.**
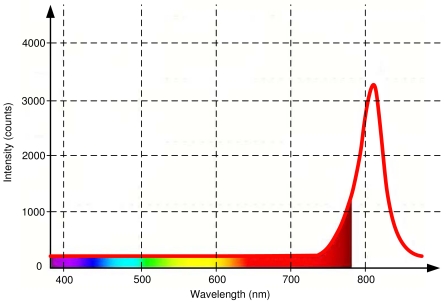
Distribution of the colors along the visible spectrum (from 390 to 780 nm approximately) along with the light emission of the selected infrared LEDs (red solid line). Notice how the emission peak of the LED is out of the visible spectrum.

Using infrared LEDs to build the pattern to be detected by the head tracking software has two main advantages. First, the infrared LEDs are not visible to the human eye. This is very important because guarantees that the visual information will not interfere with the operators' tasks. This is shown in Figure 6, where the spectrum of emission of an infrared LED centered in 810 nm is compared with the wavelength of the different colors perceived by humans. The figure shows that eye perception stops at 780 nm approximately, so an infrared emission over 810 nm will not be visible.

The second advantage is related with the use of filters, that allows to get only the infrared data in which we are interested and reduces the requirements for the detection algorithms. It is easy to see in Figure 6 that a high-pass filter centered in 780 nm will eliminate most of the visual information. Of course, the filter used in the camera has to match with the infrared characteristics of the LEDs. More details will be given in the following sections.

### 3.2. Headphones

The proposed prototype for the head tracking system makes use of the headphones of the operator. Such devices are common in control and teleoperation stations.

**Figure 7.**
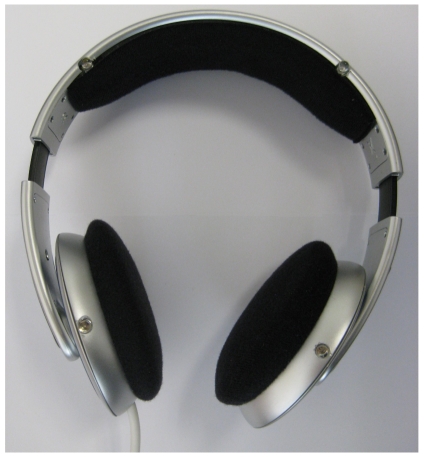
Headphones with integrated infrared LEDs.

Off-the-shelf headphones have been modified to include the infrared pattern with four LEDs (Figure 7 shows a photograph of the system). The headphones work as the usual ones, with the difference that a connector to power the LEDs has been added. In particular, this connector is an USB cable that allows taking the power directly from the computer, simplifying the setup of the system in its current prototype stage (the cable could be substituted by a set of batteries attached to the headphones).

The final size and exact shape of the infrared pattern depend on the particular head volume of the user. Thus, it is needed to measure the pattern if an accurate estimation of the motion is required. Nevertheless, experimental results show that small changes in the pattern do not cause big impacts into the final head tracking estimation. Notice that it is not needed for any interaction between the headphones and the head tracking system, i.e., the headphones are a passive system that emits infrared light and there is no information loop with the software running on the computer.

### 3.3. Visual Camera

Firewire cameras allow the modification of several parameters related to the image capture stage. In our case, three parameters are relevant to have a reliable image binarization stage: gain and brightness (set to zero) and the auto-exposure (activated to automatically adjust to changes in illumination).

**Figure 8.**
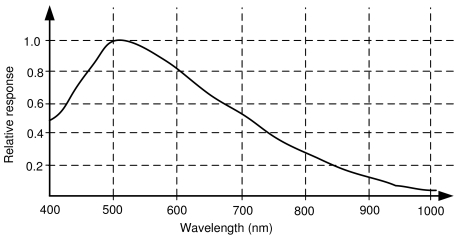
Sensitivity of the camera sensor.

On the other hand, the camera sensor must be able to get as much information as possible from the near infrared in order to detect the emission of the LEDs. The sensitivity of the sensor mounted in the camera is shown in Figure 8. It can be seen how the sensitivity is maximal around 510 nm (green colors) and how the sensitivity around the 810 nm (our infrared LEDs) is about 30%. Then, a filter to remove the visual information captured by the sensor is needed.

### 3.4. Infrared Filter

The filter should allow to cut down the information gathered by the camera, focusing into the infrared spectrum. This filter is attached to the optics of the camera and removes all the information out of the selected band. The following constraints have been taken into account:

- The filter must have as much transparency as possible around 810 nm.
- The filter should reject any visual component below 770 nm.

**Figure 9.**
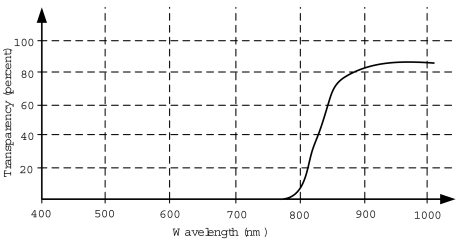
Transparency of the selected infrared filter.

Under these constraints, the filter 093 provided by *The Imaging Source* company has been selected. The transparency of the filter can be seen in Figure 9. The transparency at 810 nm is approximately 30% and it rejects all the components below 750 nm.

**Figure 10.**
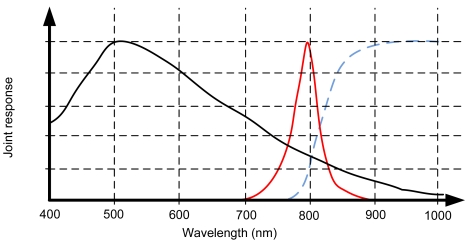
Joint response of the overall system: camera sensor (black solid line), infrared emission (red solid line) and filter transparency (blue dashed line.)

Finally, Figure 10 presents the joint response of the whole system: infrared emission, filter and camera sensor. It can be seen how the camera sensor will reject all the visual components, focusing in the near-infrared emission of the LEDs. Although there is a significant attenuation of the LEDs emission due to the filter and the sensor sensitivity, it is compensated with a higher infrared emission.

## 4. Experimental Results and Validation

In order to validate the proposed prototype of the head tracking system, several experiments have been carried out with two screens and the camera located between them. The headphones with the infrared pattern have been used to track the operator. Summarizing, the system is composed by:

- A firewire mono camera with 640 × 480 resolution and the sensitivity showed in Figure 8. The camera captures at 30 Hz.
- The camera intrinsic calibration parameters are known. Radial distortion is assumed to be negligible and was not considered in the experiments.
- The camera has an infrared filter attached with the response showed in Figure 9.
- The headphones showed in Figure 7.
- The headphones integrate four infrared LEDs with the emission response showed in Figure 6.
- The head tracking software runs in an off-the-shelf three years old Desktop PC (Intel Core2 Duo 2.33GHz processor and 2GByte of RAM).

### 4.1. Position and Orientation Experiments

**Figure 11.**
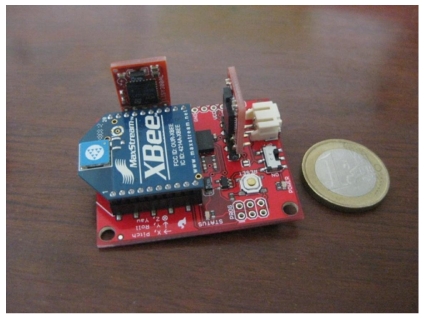
IMU used to validate the results.

Validating the estimations provided by the head-tracking system requires having another sensor able to provide a ground truth for the estimation (position and orientation). Regarding the orientation, a wireless IMU (see Figure 11) was attached to the headphones to measure the current orientation of the user's head with an accuracy of 0.5 degrees.

**Figure 12.**
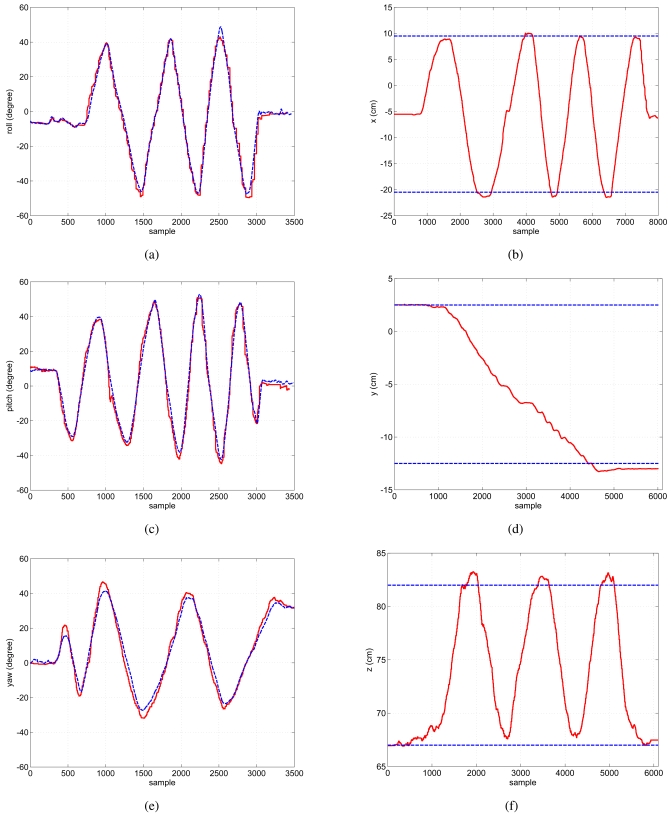
*Left*: Three different experiments with roll, pitch and yaw rotations. Dashed blue line: Ground-truth provided by the IMU. Red solid line: Head-tracking system estimation. *Right*: Three different experiments with displacements in *x̂, ŷ* and *ẑ* axes. Dashed blue line: Measured displacement. Red solid line: Head-tracking system estimation.

Figure 12 shows the estimation in orientation provided by the head tracking system. Three experiments were carried out, each of them making pure rotations in roll (a), pitch (c) and yaw (e). Figure 12 only shows the angle of interest in each of these experiments and it can be seen how the estimations follow the ground-truths with small errors, approximately of 2 degrees in mean.

The experiments in orientation show how the head-tracking system induces small delays in the estimation when the user's head quickly changes its direction, particularly in yaw (see Figure 12e). This is mainly due to the model used in the prediction stage of the Kalman Filter. Nevertheless, this behavior does not affect the global estimation of the orientation and only induces small transient errors.

On the other hand, an indoors motion capture system could be used to validate the head tracking position estimation. However, in our laboratory that system is not available, so the following setup was used: instead of moving the users's head, the infrared pattern was placed in a fixed position/orientation and the camera was attached to a small rail and moved following rectilinear trajectories in the three different axes:

- Figure 12.b: the camera was moved to left 15 cm, back to the initial position and finally to the right 15 cm again.
- Figure 12.d: the camera was moved downwards 10 cm.
- Figure 12.f: the camera was moved 15 cm perpendicular to the infrared pattern and back to the initial position.

In general, it can be seen how the estimations from the head-tracking system reach the references (blue dashed lines in the figures) with small errors of around 1 cm. It should be taken into account that the camera was moved through the rail manually, so small errors are induced into the estimation.

Finally, the processing overhead of the approach has been analyzed and some figures have been computed. As it has been pointed out in the experimental setup description, the computer used was a three years old Intel Dual Core Desktop PC. Table 1 shows the measured processing times in mean and standard deviation during the normal operation of the head tracking system. The figures have been organized according to the different head tracking stages (see Figure 3): pattern detection, pattern tracking, homography computation and motion estimation. Notice how the time required for homography and motion computation together is smaller than 40 *μs*. It is worth to mention that the tracking stage allows decreasing the computational time with respect to the pattern detection stage. Then, once the pattern has been detected, tracking is more efficient than trying to detect it again. From these results, it can be seen that this approach could work at more than 100 Hz if a high speed camera is available.

### 4.2. Tests with the Implementation of a “Virtual” Head-Mounted Display

A head-mounted display (HMD) is a display device, worn on the head or as part of a helmet, that has a small display optic in front of one (monocular HMD) or both eyes (binocular HMD). A “virtual” head-mounted display has been implemented to test and validate the proper operation of the head tracking system. The idea is to show information in the teleoperation station in such a way that this information follows the head position and orientation all along the screens of the station.

Virtual head-mounted displays could be very useful to provide critical information always in the field of view of the operator, whatever the screen at which he is looking at. This feature would help to decrease the stress of the users.

**Figure 13.**
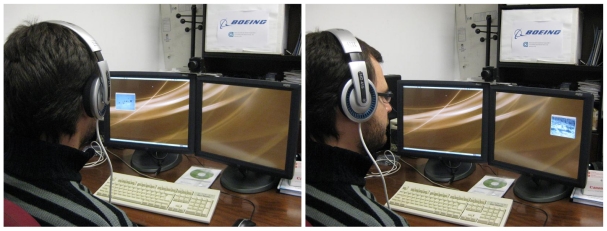
Photographs of the “virtual” head-mounted display in operation when the user looks at left and right respectively.

Thus, the information provided by the head tracking system developed has been used to implement a “virtual” head-mounted display that locates a window with information in the field of view of the user. In addition, a feature for performing zoom in the information depending on the distance of the user's head to the screen has been also included. Figure 13 shows two pictures of the system running. In this example, the information window simply shows a video with aerial images from an Unmanned Aerial Vehicle (UAV) teleoperated from the station.

During the experiments the system worked as expected, following the orientation of the head and placing the window in the field of view of the user (a video is available in http://grvc.us.es/VHMD). Nevertheless, having the information window moving over almost every possible position in the screens was not always convenient due to the risk of hiding other static critical information. To solve this drawback, a set of “virtual rails” were implemented to restrict the areas in which the information window could move. The user can place the information window in one of these rails and the window will move according to the head orientation over it.

## 5. Conclusions and Future Work

This paper shows the theoretical background, development and implementation details of a head tracking system suitable for teleoperation stations or control centers. It considers the usual specifications of those environments such as multi-screens, reliability and integration flexibility.

The system offers full 6DoF information at 30 Hz. This limit mainly comes from the camera used, i.e., if a higher-rate camera is integrated, the system could be able to work at more than 100 Hz in an off-the-shelf computer.

The paper also presented experimental results with the system prototype. Those results, compared to the ground truth employed, show small errors in the estimation. Moreover, the prototype has been also applied to implement a “virtual” Head-Mounted Display system with good results.

The setup based in one camera in front of the operator can be extended to a system with multiple cameras around. One of the limitations of the presented head-tracking system comes from both the field of view of the camera and the emission patterns of the infrared LEDs. This limitation can be overcome by using multiple cameras and fusing the information to generate a more accurate and reliable estimation. Moreover, the measurements from accelerometers and gyroscopes located on the head of the operator can be also integrated to further improve the estimation. These two improvements will be considered by the authors in future developments.

In addition, future work will also consider exploiting the homographies computed during the system operation to perform automatic intrinsic camera calibration by means of the method proposed by [13].

## Figures and Tables

**Table 1.** Measured processing overhead of the head tracking approach. Each column shows the mean and standard deviation of the required processing time expressed in microseconds.

	Detection	Tracking	Homography	Motion
mean (*μs*)	1248	1114	17	21
std. dev. (*μs*)	364	185	1	1
